# Three-Leaf-Clover Thyroid and Minimally Invasive Trans-Cervical Synchronous Thyroidectomy and Ectopic Mediastinal Thyroid Tissue Removal: Does the Age of the Patient Count amid a Multifaceted Strategy?

**DOI:** 10.3390/clinpract14060175

**Published:** 2024-10-22

**Authors:** Claudiu Nistor, Mihai-Lucian Ciobica, Oana-Claudia Sima, Anca-Pati Cucu, Mihai Costachescu, Adrian Ciuche, Lucian-George Eftimie, Dana Terzea, Mara Carsote

**Affiliations:** 1 Department 4—Cardio-Thoracic Pathology, Thoracic Surgery II Discipline, “Carol Davila” University of Medicine and Pharmacy, 0505474 Bucharest, Romania; claudiu.nistor@umfcd.ro (C.N.); adrian.ciuche@umfcd.ro (A.C.); 2 Thoracic Surgery Department, “Dr. Carol Davila” Central Emergency University Military Hospital, 010825 Bucharest, Romania; anca-pati.cucu@drd.umfcd.ro (A.-P.C.); mihaicostachescu@gmail.com (M.C.); 3 Department of Internal Medicine and Gastroenterology, “Carol Davila” University of Medicine and Pharmacy, 020021 Bucharest, Romania; 4 Department of Internal Medicine I and Rheumatology, “Dr. Carol Davila” Central Military University Emergency Hospital, 010825 Bucharest, Romania; 5 PhD Doctoral School, “Carol Davila” University of Medicine and Pharmacy, 0505474 Bucharest, Romania; 6 Department of Pathology, “Dr. Carol Davila” Central Military University Emergency Hospital, 010825 Bucharest, Romania; lucian.eftimie@unefs.ro; 7 Discipline of Anatomy and Biomechanics, National University of Physical Education and Sports, 060057 Bucharest, Romania; 8 Department of Pathology, C.I. Parhon National Institute of Endocrinology, 011863 Bucharest, Romania; danaterzea@gmail.com; 9 Department of Endocrinology, “Carol Davila” University of Medicine and Pharmacy, 020021 Bucharest, Romania; carsote_m@hotmail.com; 10 Department of Clinical Endocrinology V, C.I. Parhon National Institute of Endocrinology, 011863 Bucharest, Romania

**Keywords:** ectopic tissue, thyroid, mediastinum, thoracic surgery, endocrine surgery, aging dyspneea, Cooper retractor, cervicotomy, cervical incision, age, thyroidectomy

## Abstract

**Background:** Ectopic organ-associated conditions belong to the larger panel of developmental ailments, and among this challenging medical and surgical chapter, ectopic endocrine glands-related picture is mostly focused on the presence of the ectopic parathyroid and thyroid. Ectopic thyroid tissue within mediastinum (ETTM) stands for a less common ETT site; while, globally, less than 1% of the mediastinum masses are ETTM. **Objective:** We aim to introduce a rare case of ETTM in a senior lady to whom one-time synchronous thyroidectomy with ETT removal was successfully performed via a minimally invasive modern procedure upon cervicotomy and intra-operatory use of the Cooper thymectomy retractor. **Results:** The panel of pros and cons with respect to providing endocrine thoracic surgery for ETTM resection in a 73-year-old subject is discussed amid a PubMed search of original English-language original reports from January 2000 until 15 August 2024 in order to identify similar distinct cases (individuals of 70 years or older who underwent surgery for ETTM). **Conclusions:** 1. We propose the term “three-leaf-clover thyroid” to capture the imaging essence of having an enlargement of both (eutopic) thyroid lobes and ETTM. 2. The modern surgical approach under these circumstances provided a rapid patient recovery with a low rate of complications and a minimum hospital stay. Hence, the procedure may be expanded to older patients after a careful consideration of their co-morbidities and of the need to releasing connected complaints (e.g., a 7-month history of intermittent dyspneea was found in this case with post-operatory remission). 3. The management remains a matter of individualised decision, and age may not be a limiting factor. 4. At the present time, this case adds to the very limited number of similar published cases in the mentioned age group that we could identify (seven patients, aged between 72 and 84; male-to-female ratio of 5 to 2; the rate of malignant ETTM was 3/7); of these cases, not all were reported to have a trans-cervical approach, and none reported the use of the Cooper thymectomy retractor to help the overall surgical procedure. This innovative surgical procedure offers the advantage of avoiding a sternotomy incision which has clear functional and aesthetic implications, while the video-assisted approach allows optimal visualization of the mediastinal anatomy and safe vascular sealing under visual control, without the risk of a major hemorrhage.

## 1. Introduction

Ectopic organ-associated conditions belong to the larger panel of developmental ailments, and, among this challenging medical and surgical chapter, ectopic endocrine glands-related picture is mostly focused on the presence of the ectopic parathyroid and thyroid [1,2,3]. Ectopic thyroid tissue (ETT), generally involving 0.3 to 1 cases per 100,000 people, has numerous locations such as the tongue and neck (other than the eutopic thyroid area), and even the ovaries, kidneys, adrenals, hepatoduodenal ligament, or lungs [4,5,6,7].

ETTs within the mediastinum (ETTM) are a less common ETT site; globally, <1% of the mediastinum masses are ETTM [4,7]. A suspected thyroid malignancy in the ETTM or the need to differentiating this mass from other primary/secondary non-thyroid tumours (in this absence of the cytological/histological analysis), as well as the particular anatomic traits of the mediastinal space that are prone to compressive symptoms more common than those seen in other ETT locations, makes ETTM patients candidates for thoracic surgery, rather than remaining under lifelong conservative surveillance, but this has been a matter of precision medicine so far (since there is no unified standard protocol/guidelines) [8,9,10,11].

We aim to introduce a rare case of ETTM in a senior lady to whom one-time synchronous thyroidectomy with ETTM removal was successfully performed via a minimally invasive modern procedure upon cervicotomy, with intra-operatory use of the Cooper thymectomy retractor. The panel of pros and cons with respect to providing endocrine thoracic surgery for ETTM resection in a 73-year-old subject is discussed amid a PubMed search of original English-language original reports from January 2000 until 15 August 2024 in order to identify similar distinct cases in individuals of 70 years or older who underwent surgery for ETTM.

## 2. Pre-Surgery Assessment: A Multidisciplinary Panel

This was a 73-year-old female who had a seven-month history of intermittent dyspnea. Otherwise, she had good health while being under therapy for co-morbidities such as mild arterial hypertension, chronic venous disease, osteopenia, and lichen planus (that was intermittently treated with topical and systemic corticosteroids). The family medical history included both her sisters who were diagnosed with essential arterial hypertension in their 60s.

Her medical history highlighted that six years before her current hospitalization, an evaluation for a multinodular goiter with euthyroidism and negative thyroid autoimmunity was performed (thyroid ultrasound showed a right hypoechoic, inhomogeneous lobe of 1.8 by 1.9 by 4.8 cm, with several nodules, the largest one being a solid, isoechoic nodule of 2.1 cm, and a left lobe of 1.6 by 1.5 by 5 cm, with several micro-nodules, and a macro-nodule of 2.8 cm). She underwent a conservative approach at that point, and then she was lost to follow-up for several years.

**Figure 1:**
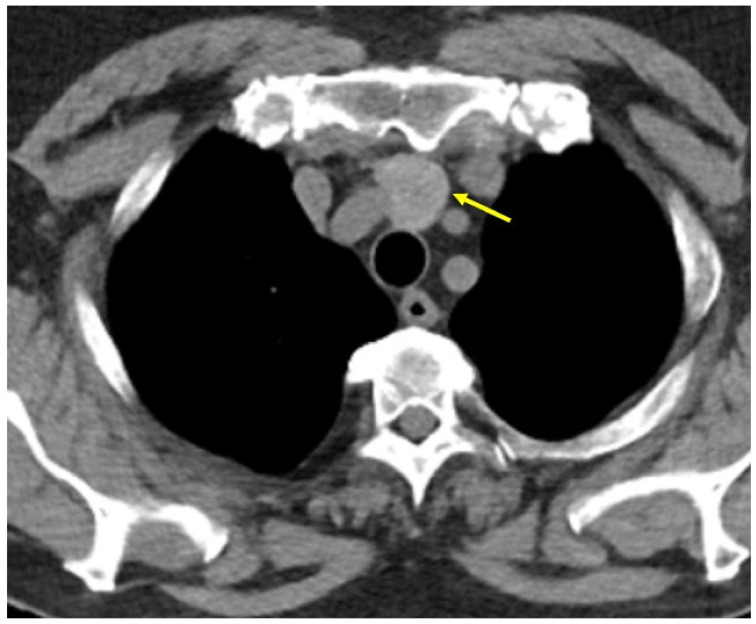
ETTM first suspicion (yellow arrow) amid contrast CT evaluation: a nodular, hyper-dense mass, with inhomogeneous iodophilia, of 2 cm (transverse diameter) by 2.5 cm (antero-posterior diameter) by 3.1 cm (cranio-caudal diameter), located on the median line of the upper mediastinum, posterior to the manubrium, anterior to the trachea, inferior to the thyroid gland and superior to the aortic arch (axial plane).

Seven months prior to current admission (while the lady started to experience some mild and intermittent breathing issues) she underwent a pneumology (including imaging) evaluation. Contrast-enhanced computed tomography (CT) showed a sub-pleural nodule of 0.5 cm in the lateral segment of the lower right lobe and a pseudo-nodular area of 1.6 by 1.4 cm, with ground glass appearance, irregular borders, and a caudal tertiary bronchus, located in the anterior segment of the upper right lobe, suggestive of underlying inflammation and infection; the trachea and main bronchi had normal aspect. Bronchoscopy and broncho-alveolar lavage revealed bronchitis, with macrophages, lymphocytes, and neutrophils, without any tumour cells. CT evaluation also revealed a nodular, hyper-dense mass, with inhomogeneous iodophilia, indicating a possible ETTM (Figure 1).

**Figure 2:**
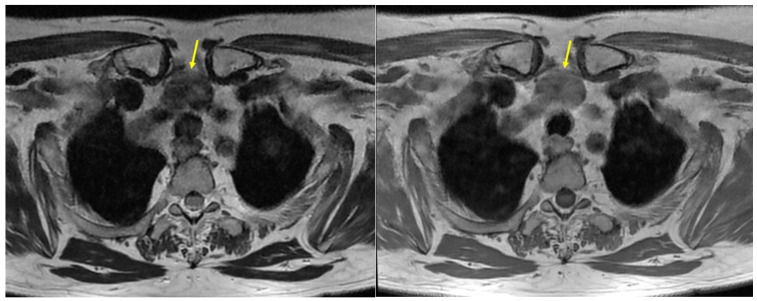
ETTM aspects (yellow arrow): MRI with IV contrast showing a nodule of 2.1 by 2.6 by 3 cm in the pre-vascular compartment of the upper mediastinum on the median line (pre-tracheal): (**left**) T2 moderate hypo-intensity and (**right**) T1 iso-intensity.

Magnetic resonance imaging (MRI) with intravenous (IV) contrast also confirmed this nodule within the mediastinum, located in the pre-vascular, pre-tracheal compartment of the upper mediastinum on the median line, under the thyroid lobes, but with clear demarcation from them, hence, highly suggesting an ETTM (Figure 2).

**Figure 3:**
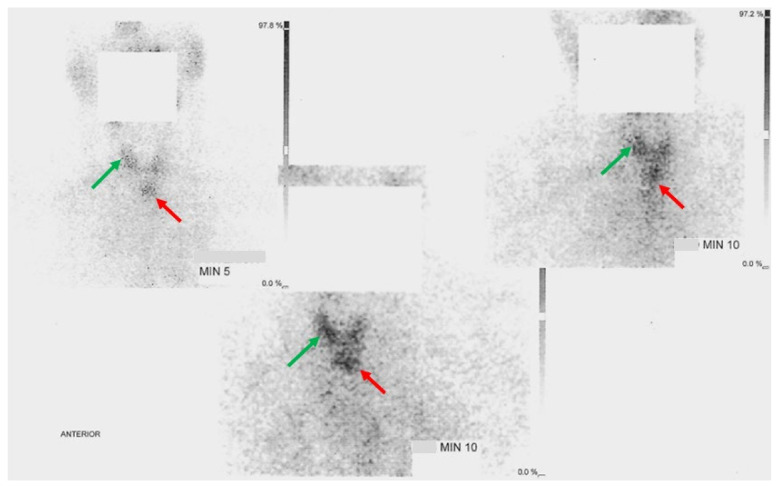
99m-Tc pertechnetate (148 MBq; effective dose 1.924 mSv) thyroid scintigraphy: an area of increased uptake in the upper part of the right thyroid lobe (orthotropic multinodular gland = green arrows) and an area of hyper-functional retrosternal thyroid tissue with hot nodules (ETTM = red arrows).

Three months prior to the current admission, she was evaluated as an outpatient. Neck ultrasound confirmed the previous aspects: the right thyroid lobe of 2.2 by 1.9 by 1.4 cm and left thyroid lobe of 1.7 by 1.6 by 3.3 cm, with an inhomogeneous pattern. The left lobe displayed an isoechoic, inhomogeneous nodular conglomerate in the medium third of 1.4 by 2.3 cm and another isoechoic, slightly inhomogeneous nodule of 1.1 by 1.6 cm in the right lobe. Further 99m-Technetium (99m-Tc) pertechnetate thyroid scintigraphy revealed a low-situated cervical (neck) thyroid gland with an area of increased uptake in the upper part of the right thyroid lobe, medium radiotracer uptake in the left lobe and isthmus, and an area of hyper-functional retrosternal thyroid tissue with hot nodules as found in ETTM (Figure 3).

**Figure 4:**
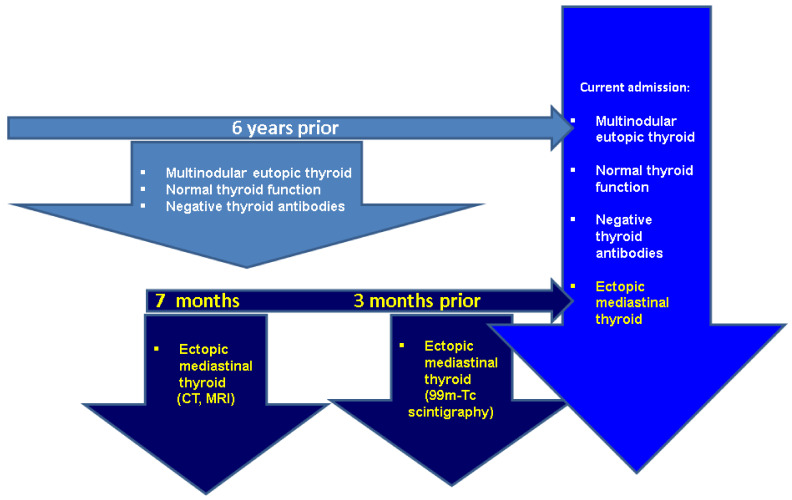
Timeline of imaging evaluation amid ETTM identification in a 73-year-old patient.

Under these circumstances, further close surveillance was recommended at that point since the patient declined any other investigations or therapy (Figure 4).

On current admission, the clinical examination was within normal limits; she had a body mass index of 35.9 kg/m^2^ and blood pressure of 124/72 mmHg under daily hypotensive medication. Biochemical evaluation showed a mild elevation of the uric acid, creatinine, and urea (a mild decrease in the glomerular filtration rate of 59 mL/min/1.73 m^2^) (Table 1).

Thyroid function was normal, with negative anti-thyroperoxidase and anti-thyroglobulin antibodies, and no primary hyperparathyroidism was confirmed. She had vitamin D deficiency and then started daily cholecalciferol replacement (Table 2).

**Figure 5:**
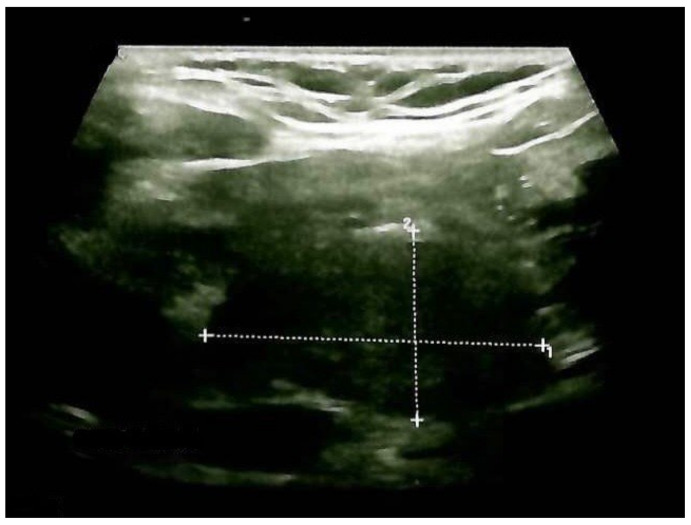
Anterior neck ultrasound: hypoechoic mass (ETTM) extending from the right thyroid lobe, with a structure similar to the thyroid and pre-tracheal extension of 3.66 by 2 by 2.6 cm (longitudinal plane).

Thyroid ultrasound confirmed previous aspects in terms of gland enlargement and a multinodular goiter with a small increase in the largest nodule diameter. The right lobe was 2 by 2 by 4.3 cm, and the left lobe was 1.8 by 1.9 by 3.8 cm, with a hypoechoic, intensely inhomogeneous pattern; the lower half of the right thyroid lobe had a nodular conglomerate of 3.1 by 1.6 by 1.7 cm. Inferior to the right thyroid lobe, a hypoechoic mass with a structure similar to that of the thyroid of 3.66 by 2 by 2.6 cm was observed (ETTM). She had no neck lymph node involvement (Figure 5).

**Figure 6:**
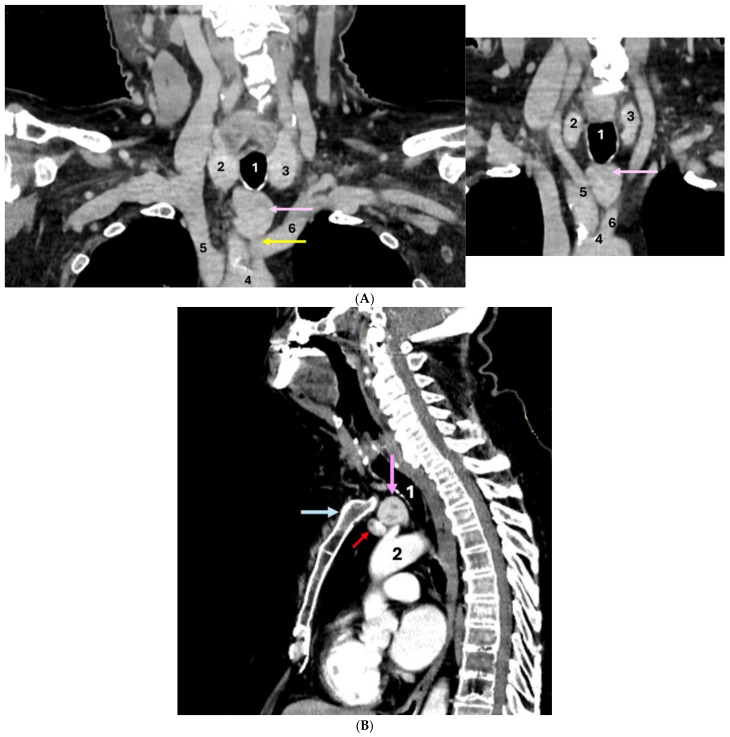
Contrast-enhanced CT scan showed: (**A**) Coronal plane (two different sections): 1—trachea; 2—right thyroid lobe; 3—left thyroid lobe; 4—ascending aorta; 5—brachiocephalic trunk; 6—left common carotid artery; ectopic thyroid (pink arrow); arterial supply (yellow arrow) for the ectopic thyroid, originating from the left common carotid artery. (**B**) Sagittal plane: 1—trachea; 2—ascending aorta; venous supply (red arrow) for ectopic thyroid, originating from the left brachiocephalic venous trunk; ectopic thyroid (pink arrow); manubrium (blue arrow).

IV contrast CT scan showed a minimally asymmetric thyroid gland and a dense, intensely iodophilic mass (ETTM) with a transverse diameter of 2.66 cm, antero-posterior diameter of 2.33 cm, and cranio-caudal diameter of 3.17 cm, located in the upper mediastinum at the level of the jugular notch, in close contact with the mediastinal blood vessels and with an imprecise cleavage plane from them. ETTM showed independent blood supply: of the arterial type coming from the left common carotid artery, respectively, of venous type, originating from the left brachiocephalic venous trunk (Figure 6).

A further cytological exam of the orthotropic thyroid was intended to be performed so that a thyroid malignancy could be ruled out, but the ultrasound-guided, fine-needle aspiration-based sample was inconsistent for a diagnosis: at the level of the nodular conglomerate in the right thyroid lobe, the colloid was in low quantity, with rare and small fibrous bands, frequent elements of chronic inflammation, small deposits of crystalloid matter, and a serous–hematic background. This was an insufficient cellular material for a cytological diagnosis (Bethesda I) [12,13,14]. A repeated fine-needle aspiration biopsy procedure was declined by the patient. A multidisciplinary decision was made to remove the ETTM due to the local compression and the particular anatomy of ETTM with regard to the other mediastinal anatomic elements, including vessels, noting the good general health status of the patient. The alternatives were taken into consideration and discussed with the patient, who agreed to an endocrine surgery.

## 3. Minimally Invasive Thoracic (Endocrine) Surgery for the Eutopic and Ectopic Thyroid

The subject underwent one-time surgical removal of the thyroid gland and the ectopic tissue through a transverse cervical Kocher incision, approximately two fingerbreadths above the sternal notch. The incision was deepened beyond the platysma, and dissection was performed at this level in the superior, inferior, and lateral directions. Any bleeding was controlled with LigaSure^®^. Crossing anterior jugular veins were sealed with LigaSure^®^ and divided. The deep cervical fascia was divided longitudinally, and the strap muscles were lifted off the right and left thyroid lobes. The right medial thyroid bundle was identified, sealed, and divided. The right recurrent laryngeal nerve was identified on the lateral aspect of the trachea and as it entered the larynx; the thyroid gland was gently separated from the nerve. The superior right thyroid vascular bundle was found, sealed, and divided.

**Figure 7:**
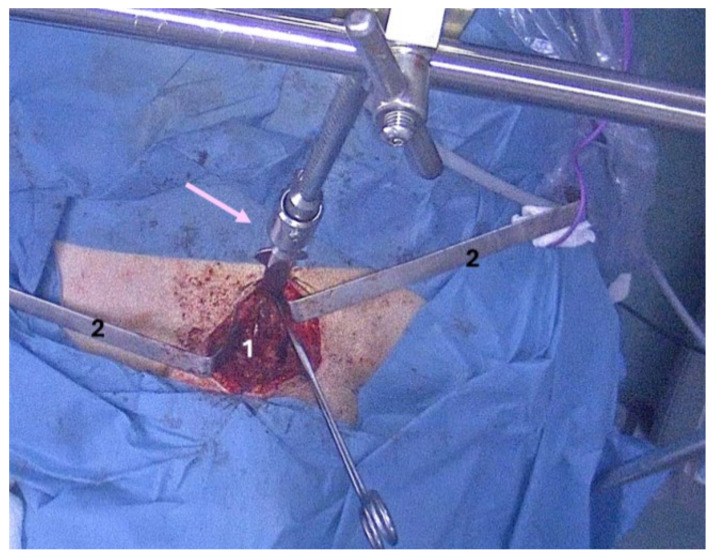
Intra-operatory capture: 1—cervical incision; 2—Farabeuf retractors; Cooper thymectomy retractor (pink arrow).

The upper right parathyroid gland was identified and left intact. The inferior thyroid vascular bundle was identified, sealed, and divided; the right inferior parathyroid gland was also identified and left intact. The left middle thyroid vein was sealed and divided. The left superior vascular bundle was found. The external branch of the superior laryngeal nerve was identified and lifted. The left thyroid lobe was gently dissected from the left recurrent laryngeal nerve, which was visualized until it approached the larynx. The left superior vascular bundle was sealed and divided. The left superior parathyroid gland was found and left intact. The left inferior vascular bundle was found, ligated with 3–0 silk, and divided. The left inferior parathyroid gland was visualized and left intact. The Cooper sternal thymus retractor with a retrosternal blade was set up, and by lifting the upper sternal part, the upper mediastinal visualization was enhanced (Figure 7).

**Figure 8:**
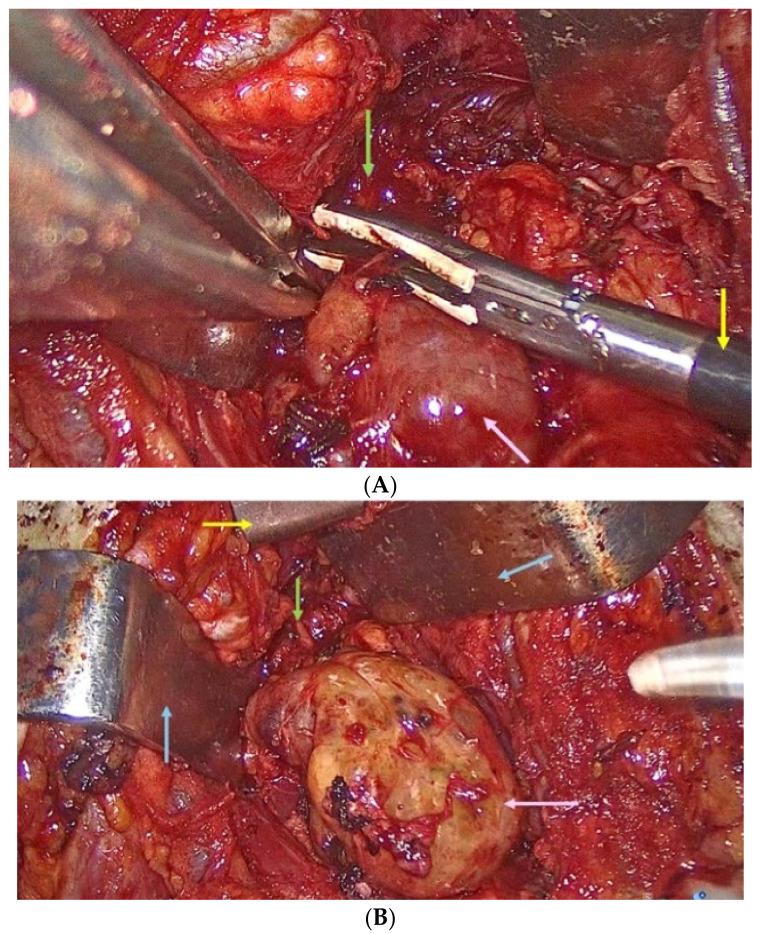
Intra- aspects: gross appearance of the upper mediastinum: (**A**) ectopic thyroid tissue (pink arrow), LigaSure sealer and divider (yellow arrow), the anterior compartment of the mediastinum (green arrow); (**B**) ectopic thyroid (pink arrow), Cooper thymectomy retractor (yellow arrow), the anterior compartment of the mediastinum (green arrow), Farabeuf retractors (blue arrows).

At the inferior pole of the thyroid, at the level of the thyroid notch, an approximately 3 cm (transverse diameter) by 3 cm (antero-posterior) by 4 cm (cranio-caudal) mass with gross appearance of thyroid tissue was identified. The vascular bundle of the mass was located at its base, connecting it to the mediastinal vessels. The inferior vascular bundle was visualized by video-assisted control. The mass was dissected from the surrounding tissues easily, and the vascular bundle was sealed with LigaSure^®^. The mass was extracted through the cervical incision. Haemostasis was achieved with LigaSure^®^. Both recurrent laryngeal nerves were re-inspected prior to closure. A thin drain tube was placed within the left neck region (Figure 8).

The strap muscles were approximated using 2-0 Vicryl^®^ suture. The skin was re-approximated with 4-0 subcuticular sutures and Steri-Strip^®^ bandages. The patient was extubated and transferred to the recovery ward. The patient tolerated well the procedure and remained stable throughout the surgery.

**Figure 9:**
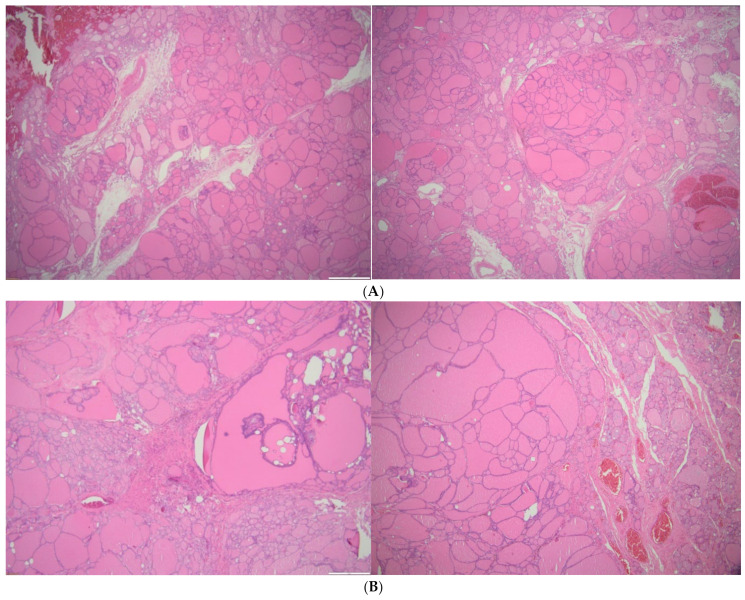
Pathological exam (microscopic aspects of ETTM; haematoxylin–eosin): aniso-follicular adenomatosis, hyperplastic epithelial areas with hyper-functional aspects, hemorrhage areas, siderophages, interstitial edema, and sclerosis. (**A**) 2×. (**B**) 4×.

Post-operatory histological evaluation of the eutopic thyroid showed a multinodular goiter with nodules of aniso-follicular adenomatosis, hyperplastic epithelial areas with hyper-functional aspects, hemorrhage areas, siderophages, partially circumscribed peri-nodular sclerosis, and small areas of interstitial lymphoid infiltrate. The macroscopic size of the right lobe was 3 by 2 cm and of the left lobe was 4 by 3 cm. ETTM was confirmed (no connective tissue with the eutopic thyroid) in terms of confirming a thyroid parenchyma with aniso-follicular adenomatosis, hyperplastic epithelial areas with hyper-functional aspects, hemorrhage areas, siderophages, interstitial edema, and sclerosis (that were similar to the eutopic neck thyroid). The macroscopic diameters of the ETTM were 3 by 4 cm (Figure 9).

**Figure 10:**
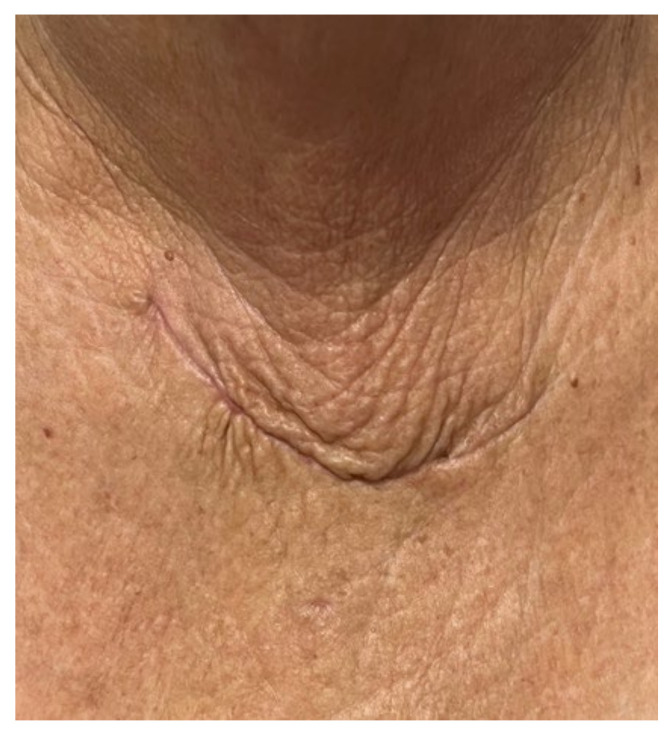
Post-operatory scar one month following one-time total thyroidectomy and ETTM removal via cervical Kocher incision.

Post-surgical follow-up confirmed a good status; the patient was released two days after the surgical procedure. Other than the iatrogenic hypothyroidism, she experienced no complications. An endocrine check-up was performed one month after thyroidectomy and ETTM excision (Figure 10).

Her biochemical profile was similar with the pre-operatory data [persistent mildly increased uric acid of 6.12 mg/dL (normal: 2.4–5.7) and creatinine of 1.01 mg/dL (normal: 0.5–0.9) mg/dL, but serum urea normalized to 34.1 mg/dL (normal: 10–50)]. Thyroid panel revealed an excessive levothyroxine substitution while the patient was treated with 100 μg of levothyroxine per day, a dose that was readjusted. She did not have hypocalcemia/hypoparathyroidism (Table 3).

**Figure 11:**
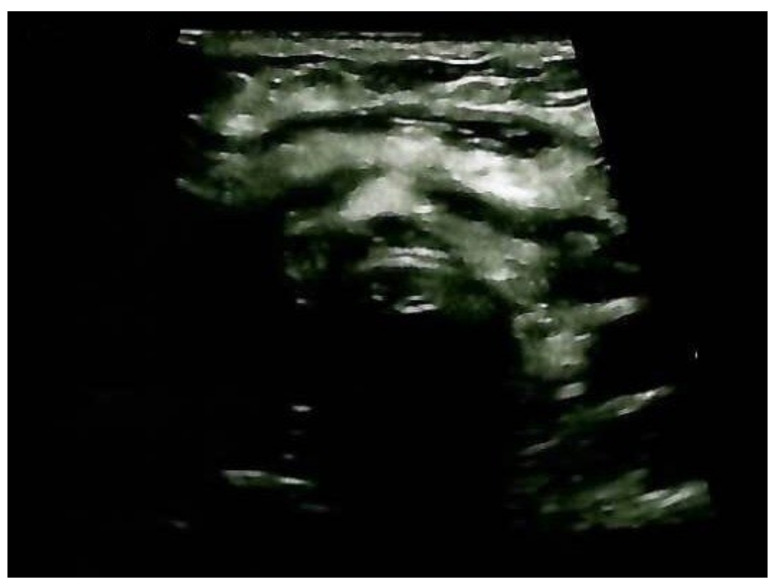
Anterior cervical ultrasonography after total thyroidectomy and ETTM removal: cervical edema and no thyroid tissue remnants.

A neck ultrasound showed mild post-surgical cervical edema and no thyroid remnants (Figure 11).

After surgery, the patient did not experience any dyspnea, and she remained asymptomatic for the following three months.

## 4. Discussion

We introduced an exceptional case of ETTM who presented unexplained dyspneea for several months that required imaging investigations; hence, a mediastinal mass was detected. The lady had a prior endocrine (thyroid) evaluation a few years before, but the index of ETTM detection was irrelevant, which is why we can hardly appreciate the rate of ectopic tissue growth under these specific circumstances. Generally, ETTM may remain asymptomatic for years unless congenital hypothyroidism or compressive complaints are detected [15]. Additionally, she experienced an increase in the multinodular goiter that might also cause intermittent breathing disturbances; thus, a synchronous removal of both thyroid tissues was taken into consideration with a post-operative good outcome and release of breathing complaints, noting the minimally invasive modern procedure [16].

Amid pre-operative imaging investigations (CT, 99m-Tc pertechnetate-based thyroid scintigraphy, and ultrasound), no connective tissue between the two thyroid locations was found (as confirmed by the pathological analysis in this case) [17,18,19]. This aspect represents a most important clue for differentiating ETTM from a substernal goiter and also to help the decision of the surgical approach in order to have the lowest rate of post-surgery complications and a reduced hospital stay [20,21,22,23].

**Figure 12:**
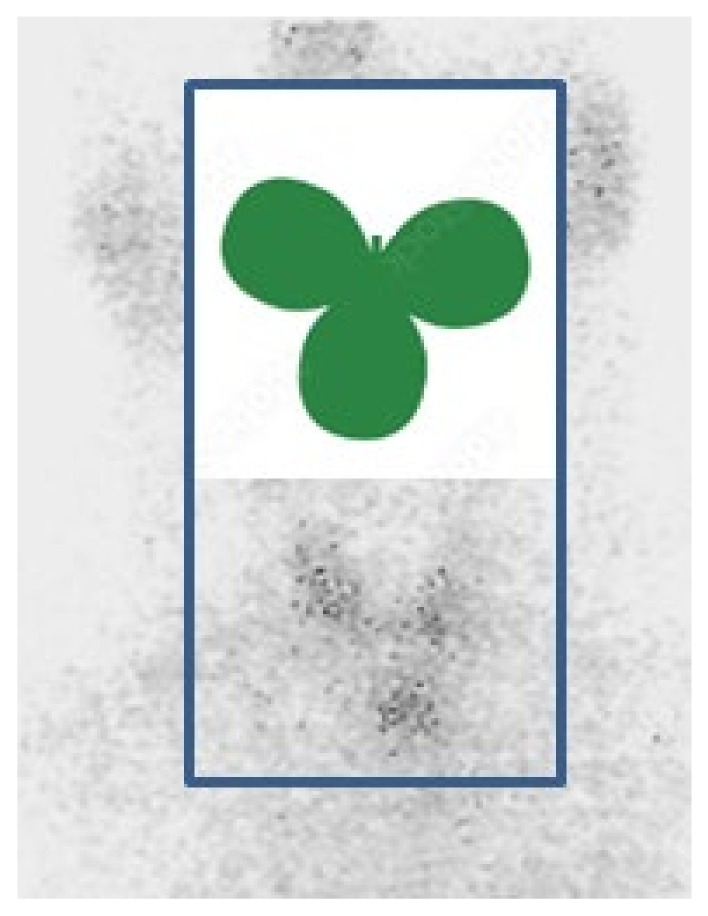
Three-leaf-clover thyroid as suggested by the two (enlarged) lobes of the neck thyroid and the presence of ETTM within the upper anterior mediastinum [24].

We propose the term “three-leaf-clover thyroid” to suggestively describe the presence of ETTM at the level of the upper anterior mediastinum in relationship with the two lobes (that might be enlarged as seen in this case) of the neck (eutopic) thyroid. If ETTM is suspected, thyroid scintigraphy may prove the most useful tool to capture the visual essence of this exceptional entity (Figure 12).

**Figure 13:**
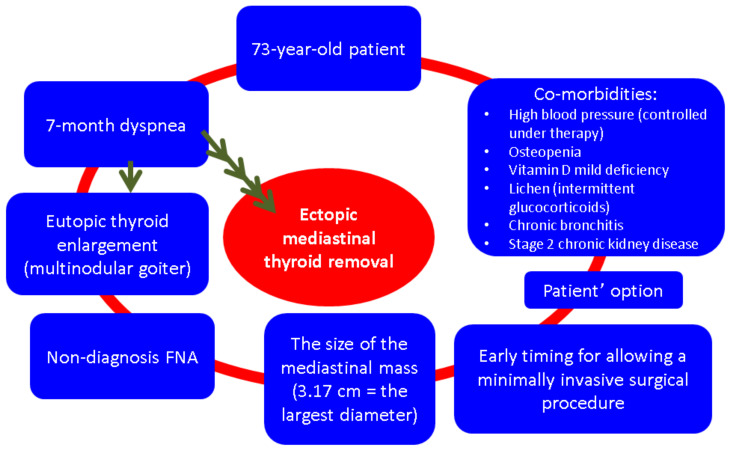
Tailored strategy amid ETTM removal in a senior patient according to our case: pros and cons to be taken into consideration with regard to the surgery decision [20,21,22,23,24,25,26,27,28,29,30,31,32,33].

Another particular aspect of this case is the decision of surgery taking into account the patient’s age and co-morbidities. Since this strategy does not represent a guideline-based protocol, a multidisciplinary team opted for ETTM and neck thyroid removal due to the associated symptoms and due to the fact that the patient was in good health. In case of an ETTM increase, the minimally invasive procedure might not be feasible or may be too risky; thus, a traditional sternotomy is required. This comes with a more complicated panel of side effects (e.g., longer operation time, increased blood loss, a higher rate of transfusion, an elevated risk of pneumonia, atelectasis, pleural effusion, and pneumothorax, etc.) that increases the overall disease burden in a senior subject; hence, the timing of the intervention should be carefully taken into consideration, too [25,26,27,28]. The presence of controlled high blood pressure, mild kidney dysfunction, and osteopenia do not represent contraindications of thoracic surgery, but their long-term primary and secondary complications might impact the morbidity and mortality in certain circumstances, and they are part of a tailored strategy in ETTM [29,30,31,32,33] (Figure 13).

As mentioned, we searched for similar cases of ETTM in subjects of 70 years or older and identified seven such cases with either benign or malign ETTM removed via different surgical approaches (aged between 72 and 84 years; male-to-female ratio of 5 to 2; the rate of malignant ETTM was 3/7) [34,35,36,37,38,39,40] (Table 4).

Of note, the first thoracoscopy-based removal by using da Vinci robot for ETTM was performed in a 72-year-old female who was confirmed with benign tissue [40]. We identified a similar case published by Mace et al. [35]: this was an 80-year-old female with an accidental MRI detection of ETTM (located at the level of the upper anterior mediastinum with tracheal deviation). A cytological report was provided by the ultrasound-guided fine-needle aspiration biopsy and showed no malignant traits. The decision was to continue with ETTM removal via cervical incision after removing the right thyroid lobe for technique purposes to safely allow ETTM resection. Following this procedure, the patient had a rapid recovery as seen in our case [35]. Notably, in our case, the decision of total thyroidectomy was mandatory based on the pre-operatory assessments, but neck thyroid removal helped the ETTM resection.

Alternatively, conservative management should be taken into account in asymptomatic patients, in cases with small ETTMs and suggestive benign features, or, quite the opposite, in subjects with aggressive malignancies underlying an overall poor prognosis [41,42,43]. For instance, Vuorisalo et al. [41] reported three patients in their 80s who underwent endobronchial ultrasound-guided fine-needle aspiration (EBUS-FNA) and then remained under surveillance [41]. Nguyen et al. [42] performed an excisional biopsy at the sternum level for metastatic anaplastic cancer in ETTM in a 90-year-old male [42]. Abdel Aal et al. [43] introduced the case of a 77-year-old female who was previously diagnosed with mammary cancer and found to have a 7 cm ETTM. The ectopic tissue was benign as confirmed upon a CT-guided percutaneous trans-thoracic core biopsy, and thus, a conservative strategy was recommended in addition to following the multimodal breast malignancy protocol [43].

This innovative surgical procedure that we described has several advantages and avoids unnecessary challenges as follows:

1. Firstly, this method avoids the partial sternotomy incision, which has clear functional and aesthetic implications. The electric sternal saw must always be present in the surgical tray.
2. Secondly, the video-assisted approach allows optimal visualization of the mediastinal anatomy and safe vascular sealing under visual control, especially in the presence of the vascular bundle on the bottom of the mass. The vascular bundle in this case originated in the left brachiocephalic venous trunk and the left common carotid, and the mass could not be delivered through the cervical incision without the risk of a major hemorrhage. The ectopic mass was located between the aortic emergence of both the common arterial trunk and the left carotid artery.
3. The arguments for an ectopic mediastinal thyroid were the lack of any parenchymal and vascular connections between the cervical thyroid and ETTM (as it also has been pre-operatory suggested amid imaging evaluation and then confirmed at pathological report).
4. The success of the procedure relied on performing a total thyroidectomy prior to removing the ectopic mass, especially given its considerable size.
5. The safety of the procedure comes from avoiding nerve damage (the left recurrent laryngeal nerve was successfully visualized from the cervical to the mediastinal end, and no relationship was found between it and the ectopic thyroid) while allowing a complete mediastinal mass removal noting that the ectopic thyroid was not invasive in the surrounding structures.

## 5. Conclusions

In our awareness, this case highlights several main key elements:

- ETTM represents an exceptional entity, and its approach is not standardized yet.
- We propose the term “three-leaf-clover thyroid” to capture the imaging essence of having an enlargement of both (eutopic) thyroid lobes as well as ETTM.
- The decision of ETTM removal takes into consideration numerous factors (patient’s co-morbidities, the profile of the eutopic thyroid, the specific anatomy of ETTM, etc.). Also, this complex panel might help the decision of choosing what kind of surgical procedure is the most adequate, a decision that is also based on the surgical team’s experience.
- A senior patient might successfully undergo a minimally invasive procedure that helps the release of the initial complaints; hence, age may not be a limiting factor in ETTM resection.
- This case adds to the very limited number of similar published cases (seven patients, with even fewer being reported to have a trans-cervical approach). At present time, we found no other publication with regard to the use of the Cooper thymectomy retractor for helping the overall surgical procedure. This surgical procedure offers the advantage of avoiding a sternotomy incision, while the video-assisted approach allows optimal visualization of the mediastinal anatomy and safe vascular sealing.

## Figures and Tables

**Table 1:** Biochemical assays on a 73-year-old female patient diagnosed with a multinodular goiter and ETTM.

Biochemical Evaluation	Patient’s Value	Normal Range
Uric acid (mg/dL)	**6.24**	2.4–5.7
Serum creatinine (mg/dL)	**1.02**	0.5–0.9
Serum urea (mg/dL)	**72**	10–50
Alanine aminotransferase (IU/L)	20	0–55
Aspartate aminotransferase (IU/L)	25	5–34
Total cholesterol (mg/dL)	139	0–200
LDL-cholesterol (mg/dL)	72.5	60–160
Triglycerides (mg/dL)	74.1	50–200
Fasting glycemia (mg/dL)	100	82–115
Glycated hemoglobin A1c (%)	5.62	4.8–5.9
Erythrocyte sedimentation rate (mm/h)	24	1–25
C-reactive protein (mg/dL)	0.3	0–0.5
Sodium (mmol/L)	140	136–145
Potassium (mmol/L)	4.8	3.5–5.1
Magnesium (mg/dL)	2	1.6–2.6

**Table 2:** Hormonal check-up in a senior lady diagnosed with ETTM according to the imaging evaluation.

Endocrine Parameter	Patient’s Value	Normal Range
TSH (Thyroid Stimulating Hormone) (μIU/mL)	0.69	0.35–4.94
FT4 (Free thyroxine) (pmol/L)	13.85	9–19
TPOAb (anti-thyroperoxidase antibodies) (IU/mL)	0.99	0–5.61
TgAb (anti-thyroglobulin antibodies) (IU/mL)	20.68	0–115
Calcitonin (pg/mL)	1.09	1–4.8
Total serum calcium (mg/dL)	9.1	8.4–10.2
Serum phosphorus (mg/dL)	3.47	2.5–4.5
PTH (parathormone) (pg/mL)	40.42	15–65
25OHD (25-hydroxyvitamin D) (ng/mL)	**17.7**	>30
Osteocalcin (ng/mL) (bone formation marker)	16.36	15–46
Alkaline phosphatase (IU/L) (bone formation marker)	58.6	35–104
P1NP (ng/mL) (bone formation marker)	34.56	20.25–76.31
CrossLaps (ng/mL) (bone resorption marker)	0.33	0.33–0.782

**Table 3:** Post-operatory endocrine panel following one-time thyroidectomy and ETTM removal in a 73-year-old lady.

Endocrine Parameter	One Month After Thoracic Surgery	Normal Range
TSH (Thyroid Stimulating Hormone) (μIU/mL) * under daily levothyroxine 100 μg daily	0.14 *	0.35–4.94
FT4 (free thyroxine) (pmol/L) * under daily levothyroxine 100 μg daily	17.08 *	9–19
Total serum calcium (mg/dL)	9.5	8.4–10.2
Serum phosphorus (mg/dL)	3.5	2.5–4.5
25OHD (25-hydroxyvitamin D) (ng/mL) ** under daily cholecalciferol 2000 IU for 2 months	29.5 **	20–100
PTH (parathormone) (pg/mL)	36.29	15–65
Alkaline phosphatase (IU/L)	56	35–104

**Table 4:** Reported cases of ETTM from January 2000 until 15 August 2024 with regard to 70-year-old patients or older who underwent thoracic surgery for ETTM removal (the display is based on the patients’ age starting with the oldest subject) (abbreviations: ETTM = ectopic thyroid tissue within mediastinum) [34,35,36,37,38,39,40].

Patient Age	Gender Distribution	Benign/Malign ETTM	Type of Thoracic Surgical Approach	Reference
84 years	male	malign (papillary carcinoma in ETTM and synchronous papillary micro-carcinoma in eutopic gland)	one-time total thyroidectomy and sternotomy	[34]
80 years	female	benign	cervical incision: one-time right lobectomy and ETTM removal	[35]
76 years	male	malign (papillary carcinoma in ETTM)	thoracotomy	[36]
74 years	male	benign	right lateral thoracotomy for excision	[37]
73 years	male	malign (papillary micro-carcinoma in ETTM)	video-assisted thoracic surgery	[38]
72 years	male	benign	right lateral thoracotomy for excision	[39]
72 years	female	benign	thoracoscopic resection (da Vinci robot)	[40]

## Data Availability

Other data are available on reasonable request.

## References

[1] Abraham B.M., Sharkey E., Kwatampora L., Ranzinger M., von Holzen U. (2023). Mediastinal Intrathymic Parathyroid Adenoma: A Case Report and Review of the Literature. Cureus.

[2] Kara C., Korkmaz H.A. (2024). Approach to Newborns with Elevated TSH: A Different Perspective from the International Guidelines for Iodine-Deficient Countries. J. Clin. Res. Pediatr. Endocrinol..

[3] Lévay B., Péter I., Fröhlich G., Koltai P., Ivády G., Járay B., Pogány P., Szőke J., Tóth E., Udvarhelyi N. (2024). Incidentally discovered ectopic tissue in the central neck region (level VI) of the neck—A case series, retrospective analysis and literature review. Magy Onkol..

[4] Zhang L., Cui X., Wang B., Du X., Hou G., Yu X. (2024). Ectopic thyroid in the hepatoduodenal ligament: A case report and literature review. Front. Oncol..

[5] Zhu C., Liu T., Yu H., Chang L., Zhang X., Yao J., Zhang G., Chen Q., He Q., Liu M. (2024). Central hyperthyroidism due to an ectopic TSH-secreting pituitary tumor: A case report and literature review. Front. Endocrinol..

[6] Nistor C., Ciuche A., Constantinescu I. (2017). Emergency surgical tracheal decompression in a huge retrosternal goiter. Acta Endocrinol..

[7] Peștean C., Pavel A., Piciu D. (2024). The Role of SPECT/CT and PET/CT Hybrid Imaging in the Management of Ectopic Thyroid Carcinoma-A Systematic Review. Diagnostics.

[8] El Haj N.I., Hafidi S., Khoaja A., Boubia S., Karkouri M., Ridai M. (2021). Ectopic mediastinal thyroid removed by U-VATS approach. A case report. Int. J. Surg. Case Rep..

[9] Di Crescenzo V., Vitale M., Valvano L., Napolitano F., Vatrella A., Zeppa P., De Rosa G., Amato B., Laperuta P. (2016). Surgical management of cervico-mediastinal goiters: Our experience and review of the literature. Int. J. Surg..

[10] Nistor C.E., Găvan C.S., Ciritel A.A., Nemes A.F., Ciuche A. (2022). The Association of Minimally Invasive Surgical Approaches and Mortality in Patients with Malignant Pleuropericarditis-A 10 Year Retrospective Observational Study. Medicina.

[11] Carsote M., Valea A., Dumitru N., Terzea D., Petrova E., Albu S., Buruiana A., Ghemigian A. (2016). Metastases in daily endocrine practice. Arch. Balk. Med. Union.

[12] Alshaikh S., Harb Z., Aljufairi E., Almahari S.A. (2018). Classification of thyroid fine-needle aspiration cytology into Bethesda categories: An institutional experience and review of the literature. Cytojournal.

[13] Cibas E.S., Ali S.Z. (2017). The 2017 Bethesda System for Reporting Thyroid Cytopathology. Thyroid.

[14] Hall E.A., Hartzband P., VanderLaan P.A., Nishino M. (2023). Risk stratification of cytologically indeterminate thyroid nodules with nondiagnostic or benign cytology on repeat FNA: Implications for molecular testing and surveillance. Cancer Cytopathol..

[15] Moran C., Schoenmakers N., Visser W.E., Schoenmakers E., Agostini M., Chatterjee K. (2022). Genetic disorders of thyroid development, hormone biosynthesis and signalling. Clin. Endocrinol..

[16] Khan N.S., Zhang Y., Bollig K., Bollig C.A. (2024). Extracervical Approaches to Substernal Thyroid Goiter Resection: A Systematic Review and Meta-Analysis. OTO Open.

[17] Anghel A., Stanciu S., Ciobica M.L., Stoicescu D., Muresan M.M. (2011). Contrast-enhanced ultrasound-clinical applications. Rom. J. Mil. Med..

[18] Stanciu S., Enciu C., Raduta I., Stoicescu D., Anghel A., Anghel D., Olan B., Ciobica L. (2016). The role of contrast-enhanced ultrasound in risk assessment of carotid atheroma. Rom. J. Mil. Med..

[19] Linhares S.M., Scola W.H., Remer L.F., Khan Z.F., Nguyen D.M., Lew J.I. (2022). Depth of mediastinal extension can predict sternotomy need for substernal thyroid goiters. Surgery.

[20] Nankee L., Chen H., Schneider D.F., Sippel R.S., Elfenbein D.M. (2015). Substernal goiter: When is a sternotomy required?. J. Surg. Res..

[21] White M.L., Doherty G.M., Gauger P.G. (2008). Evidence-based surgical management of substernal goiter. World J. Surg..

[22] Unlu M.T., Aygun N., Kostek M., Isgor A., Uludag M. (2022). Substernal Goiter: From Definitions to Treatment. Med. Bull. Sisli Etfal Hosp..

[23] Uludag M., Unlu M.T., Aygun N., Isgor A. (2022). Surgical Treatment of Substernal Goiter Part 2: Cervical and Extracervical Approaches, Complications. Med. Bull. Sisli Etfal Hosp..

[24] https://stock.adobe.com/ro/search?k=three+leaf+clover&asset_id=77962996.

[25] Linhares S.M., Scola W.H., Remer L.F., Farrá J.C., Lew J.I. (2022). Morbidity Associated with Surgical Removal of Substernal Thyroid Goiters. J. Surg. Res..

[26] Battistella E., Pomba L., Sidoti G., Vignotto C., Toniato A. (2022). Retrosternal Goitre: Anatomical Aspects and Technical Notes. Medicina.

[27] Brenet E., Dubernard X., Mérol J.C., Louges M.A., Labrousse M., Makeieff M. (2017). Assessment and management of cervico-mediastinal goiter. Eur. Ann. Otorhinolaryngol. Head Neck Dis..

[28] Tsilimigras D.I., Patrini D., Antonopoulou A., Velissaris D., Koletsis E., Lawrence D., Panagiotopoulos N. (2017). Retrosternal goitre: The role of the thoracic surgeon. J. Thorac. Dis..

[29] Zenoaga-Barbăroșie C., Berca L., Vassu-Dimov T., Toma M., Nica M.I., Alexiu-Toma O.A., Ciornei C., Albu A., Nica S., Nistor C. (2023). The Predisposition for Type 2 Diabetes Mellitus and Metabolic Syndrome. Balk. J. Med. Genet..

[30] Nakaya M., Ito A., Mori A., Oka M., Omura S., Kida W., Inayoshi Y., Inoue A., Fuchigami T. (2017). Surgical treatment of substernal goiter: An analysis of 44 cases. Auris Nasus Larynx.

[31] McKenzie G.A., Rook W. (2014). Is it possible to predict the need for sternotomy in patients undergoing thyroidectomy with retrosternal extension?. Interact. Cardiovasc. Thorac. Surg..

[32] Riffat F., Del Pero M.M., Fish B., Jani P. (2013). Radiologically predicting when a sternotomy may be required in the management of retrosternal goiters. Ann. Otol. Rhinol. Laryngol..

[33] Qureishi A., Garas G., Tolley N., Palazzo F., Athanasiou T., Zacharakis E. (2013). Can pre-operative computed tomography predict the need for a thoracic approach for removal of retrosternal goitre?. Int. J. Surg..

[34] Toda S., Iwasaki H., Suganuma N., Okubo Y., Hayashi H., Masudo K., Nakayama H., Masuda M. (2020). Occult Thyroid Carcinoma without Malignant Thyroid Gland Findings during Preoperative Examination: Report of Three Cases. Case Rep. Endocrinol..

[35] Mace A.D., Taghi A., Khalil S., Sandison A. (2011). Ectopic sequestered thyroid tissue: An unusual cause of a mediastinal mass. ISRN Surg..

[36] Hu J., Li M., Xu L. (2017). Ectopic thyroid cancer diagnosed by endobronchial ultrasound-guided transbronchial needle aspiration. Thorac. Cancer.

[37] Karapolat S., Bulut I. (2008). Ectopic Posterior Mediastinal Thyroid: A Case Report. Cases Journal.

[38] Caroço T.V., Saraiva R.P., Baião J.M., Nogueira T., Garcia A.L., Costa Almeida C.E. (2023). Mediastinal papillary thyroid carcinoma treated by video-assisted thoracic surgery—Case report. Int. J. Surg. Case Rep..

[39] Pilavaki M., Kostopoulos G., Asimaki A., Papachristodoulou A., Papaemanouil S., Palladas P. (2009). Imaging of Ectopic Intrathoracic Multinodular Goiter with Pathologic Correlation: A Case Report. Cases Journal.

[40] Bodner J., Fish J., Lottersberger A.C., Wetscher G., Schmid T. (2005). Robotic resection of an ectopic goiter in the mediastinum. Surg. Laparosc. Endosc. Percutan. Tech..

[41] Vuorisalo A., Tommola E., Eloranta P., Vanhelo T., Paavonen T., Kholová I. (2023). Ectopic thyroid in EBUS: Experience from a quality assurance programme. APMIS.

[42] Nguyen D., Htun N.N., Wang B., Lee B., Johnson C. (2023). An Anaplastic Thyroid Carcinoma of the Giant-Cell Type from a Mediastinal Ectopic Thyroid Gland. Diagnostics.

[43] Abdel Aal M., Scheer F., Andresen R. (2015). Ectopic mediastinal thyroid tissue with a normally located thyroid gland. Iran. J. Radiol..

